# Impact of excess gestational and post-weaning energy intake on vascular function of swine offspring

**DOI:** 10.1186/s12884-014-0405-z

**Published:** 2014-12-12

**Authors:** Pardis Taheripour, Mark A DeFord, Emily J Arentson-Lantz, Shawn S Donkin, Kolapo M Ajuwon, Sean C Newcomer

**Affiliations:** Department of Health and Kinesiology, Purdue University, 3705 Chancellor Way, West Lafayette, IN 47906 USA; Interdepartmental Nutrition Program, Purdue University, West Lafayette, IN USA; Department of Animal Science, Purdue University, West Lafayette, IN USA; Department of Kinesiology, California State University San Marcos, San Marcos, CA USA

**Keywords:** Gestational diet, Postnatal diet, High-energy diet, Fetal programming, Atherosclerosis

## Abstract

**Background:**

The development of long-term vascular disease can be linked to the intrauterine environment, and maternal nutrition during gestation plays a critical role in the future vascular health of offspring. The purpose of this investigation was to test the hypothesis that a high-energy (HE) gestational diet, HE post-weaning diet, or their combination will lead to endothelial dysfunction in offspring.

**Methods:**

Duroc × Landrace gilts (n = 16) were assigned to either a HE (10,144 Kcal/day, n = 8) or normal energy (NE: 6721 Kcal/day, n = 8) diet throughout pregnancy. Piglets were placed on either a NE or HE diet during the growth phase. At 3 months of age femoral arteries were harvested from offspring (n = 47). Endothelial-dependent and -independent vasorelaxation was measured utilizing wire-myography and increasing concentrations of bradykinin (BK) and sodium nitroprusside (SNP), respectively.

**Results:**

BK and SNP induced vasorelaxation were significantly reduced in the femoral arteries of gestational HE offspring. However, no effect for the post-weaning diet on BK and SNP induced vasorelaxation was seen. This investigation demonstrates that a HE diet prenatally diminishes both BK and SNP induced vasorelaxation in swine.

**Conclusions:**

These findings suggest that a HE gestational diet can play a critical role in the development of offspring’s vascular function, predisposing them to endothelial dysfunction. This dysfunction may lead to atherosclerotic disease development later in life.

## Background

Throughout the past few decades, obesity has become increasingly prevalent [1]. This obesity pandemic, specifically in those of childbearing age, has created a significant burden for the health care system [1]. For instance, in the United States, between 18% and 35% of women are considered clinically obese during their pregnancy [1]. Furthermore, 40-60% of pregnant women exceed the weight gain recommendations set forth by the Institute of Medicine (IOM) [2].

Over-nutrition, in combination with an inactive lifestyle, is the main cause of excess weight gain during pregnancy [3]. Excess maternal weight gain is known to have various negative outcomes for the developing offspring. For instance, over-nutrition/obesity during pregnancy can lead to obesity, insulin resistance, and hypertension in the offspring [4-8]. It has also been reported that over-nutrition during pregnancy increases the incidence of cardiovascular disease in the offspring [9,10]. Endothelial dysfunction, as manifested by reduced nitric oxide (NO) bioavailability, is a well-known precursor to cardiovascular disease [11,12]. Consequently, high fat gestational diets have been reported to produce reductions in endothelial function in the offspring [13-15]. Interestingly, this endothelial dysfunction reported with high fat gestational diets has not been associated with decreases in endothelial nitric oxide synthase (eNOS) gene expression in the offspring [14]. It is important to note that these results must be interpreted cautiously given the relatively small number of studies and limited animal models utilized [13-15].

Although it is critical to assess how maternal over-nutrition effects offspring, evaluating the impact of a postnatal diet is equally as important given the plasticity of organ systems during early child development. It has been established that postnatal diets can alter the outcomes of gestational diets by exacerbating hypercholesterolemia, hypertension, glucose and lipid metabolism, and body weight in offspring [7,16]. To date, though, only a few studies have examined the impact of both a prenatal and postnatal diet on the vascular function of offspring. The results of these studies were equivocal, with one study [14] reporting improved endothelial function and the other study [15] reporting reduced endothelial function when offspring exposed to high fat diet prenatally were placed on a normal diet during postnatal life. Moreover, a recent study demonstrated that exposure to high fat prenatal or postnatal diets similarly decreased vascular function [17]. Given that obesity has become increasingly prevalent amongst women of childbearing age [1], it is critical to determine if a postnatal diet can modulate the effects of a high-energy gestational diet. This forthcoming information could be of great clinical significance and thus more research is needed to clarify previous findings.

Therefore, the aim of this investigation was to determine the impact of excessive maternal and post-weaning energy intake on offspring vascular function in a large animal swine model. A high-energy as opposed to high-fat diet was chosen as it provides a more realistic comparison to a human diet. We hypothesized that exposure to a high-energy (HE) gestational diet, HE post-weaning diet, or a combination of the two will lead to decreased endothelial function.

## Methods

### Animal model

Swine (Durocs × Landrace) were the chosen model for this experiment because of their cardiovascular similarities to humans [18] and their previous utilization in fetal programming experiments [19]. All animal procedures were carried out with prior approval from the Purdue University Animal Care and Use Committee.

### Gestational protocol

Healthy gilts selected from the Purdue Research Farm herd (n = 16) were artificially inseminated and randomly assigned to either a HE (3.1 kg feed/day, n = 8) or normal energy (NE: 2.05 kg feed/day, n = 8) diet once pregnancy was confirmed. Gilts were individually housed in gestation stalls and limit fed (HE: 10,144 Kcal/day vs. NE: 6,721 Kcal/day) the same diet throughout pregnancy with ad libitum access to water. The NE diet was formulated to meet energy needs of pregnant gilts as recommended by the National Research Council Requirements for Swine. The HE diet was formulated to induce excess pregnancy weight gain by a 50% increase in metabolizable energy. Protein was matched as closely as possible on a grams per day basis for both HE and NE groups to prevent differences in weight gain due to an increase in muscle mass. Consequently, the increase in calories was attained by increasing both calories from fat and calories from carbohydrates. Commercially available ingredients were utilized in the generation of both the HE and NE diets (Table 1). All sows received the same lactation diet for ad libitum intake. Sows were weighed at baseline, at months 1, 2, and 3 of gestation, and at the end of lactation. The procedures for animal care treatment diets are part of a more extensive experiment that is detailed elsewhere (Arentson, E. J. Fetal programming: Maternal fructose consumption, exercise and gestational weight gain. Purdue University). ProQuest Dissertations and Theses http://search.proquest.com/docview/1237150789, 2012). Thus data presented here for birth weight and offspring growth is derived from a larger investigation.

**Table 1:** **Ingredients of gestational diets**

	**Maternal diets**		
**Ingredient**	**Normal energy** ^**5**^	**High energy** ^**6**^	**Lactation**
Corn, g/kg	52.80	74.00	60.90
Soybean meal, g/kg	12.20	6.50	24.10
Dried Distillers Grains, g/kg	30.0	15.0	7.5
Choice White Grease, g/kg	1.0	1.65	3.0
Limestone, g/kg	1.55	1.06	1.44
MonoCal, g/kg	0.75	0.50	1.42
Swine Vitamin Premix^1^, g/kg	0.25	0.17	0.25
Sow Vitamin Premix^2^, g/kg	0.25	0.17	0.25
Selenium 600 Premix^3^, g/kg	0.05	0.035	0.05
Total Mineral Premix^4^, g/kg	0.125	0.85	0.125
Phytase, g/kg	0.10	0.10	0.10
Salt, g/kg	0.50	0.35	0.50
Rabon Larvacide, g/kg	0.13	0.13	0.13
Diffusion Plus, g/kg	0.25	0.25	0.25
Feed intake, kg/day	2.05	3.00	ad lib
Protein intake, g/day	370	395	ND^7^
Lysine intake g/day	16.03	15.8	ND^7^
Carbohydrate intake, g/day	741	1457	ND^7^
Metabolizable Energy intake, kcal/day	6761	10144	ND^7^

^1^Purdue Swine Vitamin Premix; Vitamin A, 544,680 IU/kg; Vitamin D3, 54,448 IU/kg; Vitamin E, 3631 IU/kg; Memdione (Vitamin K), 182 mg/kg.

^2^Sow Vitamin Premix: KSU Sow Vitamin Add Pack with CarniChrome from ADM (Biotin,18.1 mg/kg; Folic Acid, 136 mg/kg; Choline, 45, 390 mg/kg; Pyridoxine, 409 mg/kg; Vitamin E, 1,816 IU/kg; Chromium, 16.3 mg/kg; Carmium, 16.3 mg/kg; Carnitine, 4,805 mg/kg).

^3^Selenium-600 premix; Calcium, 28-31%, Selenium, 0.06% equivalent to 123.6 mg/kg.

^4^Purdue Non-Sulfur Trace Mineral Premix: Iron, 51.05%; Zinc, 20.73%; Manganese, 2.86%; Copper, 1.56%; Iodine, 0.046%.

^5^Diet Used for NE treatment during gestation. ^6^Diet used for HE treatment during gestation.

^7^Not determined, lactation diet was fed for ad libitum intake.

### Post-weaning protocol

At birth, piglets were not cross-fostered throughout the suckling period. Offspring were weighed 48 hours after birth, the last day of weaning, and at 3 months of age. Male and female littermates were blocked by weight and maternal diet and assigned, within litter, to either a post weaning normal energy or post weaning high-energy treatment. At six weeks of age, offspring were transitioned to a phase four nursery diet (either HE or NE), which was continued until three months of age when they were sacrificed for vascular functional experiments (Table 2).

**Table 2:** **Ingredients of offspring diets**

	**Weaning to 4 weeks**	**4 to 6 weeks**		**6 to 12 weeks**	
**Ingredient**	**All pigs**	**PWnNE**	**PWnHE**	**PWnNE**	**PWnHE**
Corn, g/kg	32.25	32.25	37.35	60.98	52.98
Soybean Meal, g/kg	13.72	13.72	19.00	24.64	24.64
Dried distillers grains, g/kg	-	-	-	7.50	7.50
Soybean Oil, g/kg	5.00	5.00	5.00	-	5.00
Limestone, g/kg	0.72	0.72	0.61	1.35	1.35
MomoCal Phosphate,g/kg	0.53	0.53	0.75	0.74	0.74
Swine Vitamin Premix^1^, g/kg	0.25	0.25	0.25	0.25	0.25
Swine Total Mineral Premix^2^, g/kg	0.12	0.12	0.12	0.12	0.12
Selenium 600 Premix^3^, g/kg	0.05	0.05	0.05	0.05	0.05
Dried Whey, g/kg	25.0	25.0	25.0		
Lactose, g/kg	5.00	5.00			
Fish Meal, g/kg	4.00	4.00	4.00		
Phytase (600 PU/g), g/kg,	0.10	0.10	0.10	0.10	0.10
Salt, g/kg	0.25	0.25	0.25	0.35	0.35
Blood meal, g/kg	6.50	6.50	3.75	-	-
Zinc Oxide, g/kg	0.37	0.37	0.37	-	-
Soy Concentrate, g/kg	4.00	4.00	2.50	-	-
Carbadox (10 g/lb), g/kg	0.25	0.25	0.25	1.00	1.00
Lysine-HCl, g/kg	0.11	0.11	0.25	0.40	0.40
DL-Methionine, g/kg	0.20	0.20	0.22	0.12	0.12
L-Threonine, g/kg	0.04	0.04	0.12	0.16	0.16
L-Tryptophan, g/kg	-	-	0.02	0.30	0.30
Rabon Larvacide, g/kg	0.025	0.025	0.25	0.25	0.25
Bansmith dewormer, g/kg	-	-	-	0.10	0.10
Copper Sulfate, g/kg	-	-	-	0.07	0.07
Crude Sulfate, g/kg	23.96	23.96	22.8	19.40	17.9
Total Lysine, %	1.73	1.73	1.65	1.29	1.21
Metabolizable Energy, kcal/kg	3545	3545	3522	3395	3771

^1^Purdue Swine Vitamin Premix: Vitamin A, 544,680 IU/kg; Vitamin D3, 54, 448 IU/kg; Vitamin E, 3631 IU/kg; Mendione (Vitamin K), 182 mg/kg; Vitamin B12, 3.2 mg/kg; Riboflavin, 726 mg/kg; d-Pantothenic Acid, 1,816 mg/kg; Niacin, 2723 mg/kg.

^2^Pudue Non-Sulfur Trace Mineral Premix: Iron, 51.05%; Zinc, 20.73%; Manganese, 2.86%; Copper, 1.56%; Iodine, 0.046%.

^3^Selenium 600-Premix: Calcium, 28-31%; Selenium, 0.06% equivalent to 123.6 mg/kg.

### Vascular experiments

At three months of age, the femoral artery was harvested from male and female offspring, representing each combination of maternal and post-weaning diet (n = 47). Due to high susceptibility to atherosclerotic disease development and use in previous research, the femoral artery was chosen for the assessment of vascular function [20,21]. Femoral arteries were cleaned of connective tissue and cut into ~3 mm sections for *in vitro* vascular function experiments. Arterial rings were measured for axial length and inner/outer diameter using a stereomicroscope (PZMIII, World Prescision Instruments, Sarasota, FL, USA) in combination with Image J software (NIH, Bethesda, MD, USA). Femoral arterial segments were then mounted on a myograph (MyobathII, World Precision Instruments, Sarasota, FL, USA) by positioning two stainless steel wires in the lumen of the vessel ring. These wires were connected to a force transducer to measure force and stretch. Arterial rings were placed in a 20 mL bath of Krebs bicarbonate solution that was heated to 37°C and bubbled with a 95% O2 and 5% CO2 gas mixture. Arteries were set to a resting tension of 8 g, which based on previous experiments, has been proven to be the optimal length-tension point for swine arteries of similar size [21]. All rings were pre-constricted using prostaglandin F2α (PGF2α; 30 μM) and allowed to reach tension equilibrium. This dose of PGF2α has been reported to produce steady-state tension ≥50% of that exhibited in response to maximal doses (10^−4^ M) of NE [22]. Endothelium-dependent, dose-dependent vasorelaxation was then assessed using cumulative addition of bradykinin (BK; 10^−11^ – 10^−6^ M), which causes the synthesis and release of nitric oxide, prostacyclin, and endothelial derived hyperpolarizing factor from endothelial cells. After rinsing and again pre-constricting with PGF2α, endothelium-independent, dose-dependent vasorelaxation was assessed using cumulative addition of sodium nitroprusside (SNP; 10^−10^ – 10^−4^ M), which is used to assess the responsiveness of vascular smooth muscle to exogenous nitric oxide.

### Statistical analysis

An analysis of variance (ANOVA) with fixed effects for treatment, time of gestation, and the corresponding interaction was used to determine the effect of gestation and lactation diet on maternal weight gain using proc glimmix in SAS 9.2 (SAS Institute, Cary, NC, USA). A Student’s *t*-test was utilized to compare differences in maternal weight gain during gestation. A Student’s *t*-test was also used to observe the maternal weight differences from the beginning to end of the lactation period. Furthermore, a Student’s *t*-test was utilized to compare differences in litter weights. Two dam’s offspring were excluded as outliers when analyzing the offspring weights. Significance for all comparisons was determined with α = 0.05. All data are represented as mean ± SE.

Results for the in-vitro myography, including both bradykinin and sodium nitroprusside experiments, were analyzed using ANOVA with fixed effects for concentration, treatment, age, and all interactions. To reduce the effect of potential correlations between offspring within the same litter, gilt was nested in treatment and piglet was nested in gilt. Least squares means in proc glimmix in SAS 9.2 (SAS Institute, Cary, NC, USA) with Tukey adjustment for multiple testing was used to establish significant differences between offspring exposed to a HE gestational diet, NE gestational diet, HE post-weaning diet, NE post-weaning diet, and all possible combinations of the four as well as differences between gender. BK and SNP data are expressed as a percent relaxation on the y-axis and log of concentration on the x-axis. “0%” represents the PGF2α-induced vasoconstriction and “100%” represents baseline tension. Significance for all comparisons was determined with α = 0.05. All data are represented as mean ± SE.

## Results

### Maternal and offspring weights

There were no differences in weight between the NE and HE groups at any point during gestation or lactation. However, significant differences were observed for total weight gain from baseline to the end of pregnancy (Table 3). Specifically, sows placed on the HE diet gained significantly more weight than sows on the NE diet. This excess maternal weight gain in the HE sows was not associated with a significant difference in offspring weight at birth. Offspring weights were also not altered by the NE or HE gestational or post-weaning diets through three months of age (Table 4).

**Table 3:** **Impact of maternal diet on gestational weight gain**

	**Maternal weights**		
	**NE**	**HE**	**p value**
Baseline (kg)	156.08 ± 5.77	155.38 ± 6.11	0.935
1 month (kg)	170.98 ± 5.00	175.8 ± 5.54	0.535
2 month (kg)	177.49 ± 4.82	184.08 ± 5.24	0.379
3 month (kg)	185.17 ± 5.05	195.21 ± 5.31	0.204
Gestational weight gain (kg)	29.08 ± 1.79	39.83 ± 3.19	<0.05*
Lactation (kg)	166.71 ± 6.40	183.2 ± 6.55	0.090

Average weight of normal energy (NE) and high energy (HE) sows across gestation and lactation. Data expressed as means ± standard errors. *Significant differences were observed for total weight gain between the NE and HE groups (P < 0.05).

**Table 4:** **Impact of post-suckling diet on offspring weight gain**

	**Offspring weights**				
	**NE/NE**	**NE/HE**	**HE/NE**	**HE/HE**	**p value**
Weaning (kg)	5.16 ± 0.40	4.98 ± 0.37	5.25 ± 0.30	5.76 ± 0.37	0.491
3 month (kg)	36.46 ± 1.96	33.11 ± 2.19	35.79 ± 2.00	34.67 ± 1.65	0.633
Post-suckling weight gain (kg)	31.29 ± 1.62	28.08 ± 1.89	30.59 ± 1.77	28.89 ± 1.38	0.494

Average weight of normal-energy and high-energy offspring from weaning to three months of age. Data expressed as means ± standard errors.

### Vessel characteristics

There were no significant differences in artery lengths, diameters, or tensions between offspring of NE and HE gilts during in vitro vascular experiments (Table 5).

**Table 5:** **Vessel characteristics**

**Vessel characteristics**	**HE/HE**	**HE/NE**	**NE/HE**	**NE/NE**
Artery Length (mm)	3.10 ± 0.14	3.06 ± 0.12	3.02 ± 0.12	2.95 ± 0.15
Artery Outer Diameter (mm)	3.32 ± 0.15	3.32 ± 0.13	3.38 ± 0.11	3.45 ± 0.13
Artery Inner Diameter (mm)	1.81 ± 0.10	1.72 ± 0.16	1.92 ± 0.15	2.01 ± 0.15
Resting Tension (g)	8.31 ± 0.14	8.14 ± 0.11	8.26 ± 0.13	8.19 ± 0.11
PGF Tension (g)	31.71 ± 2.86	32.83 ± 1.55	30.46 ± 0.18	31.11 ± 1.47

Vessel characteristics of normal energy (NE) and high energy (HE) offspring at three months of age. Resting tension is the amount of tension before preconstriction with PGF2α (30 μM). PGF tension corresponds to PGF2α induced tension before adding cumulative doses of BK and SNP. Data expressed as means ± standard errors. No significant differences were observed (α < 0.05).

### Vascular function

**Figure 1:**
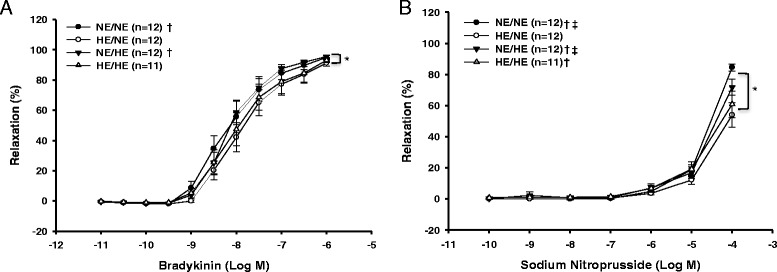
**Endothelium-dependent (A) and endothelium-independent (B) relaxation responses to increasing doses of BK and SNP in the femoral artery of offspring exposed to gestational NE or HE diets and post-weaning NE or HE diets, at three months of age.** (†) Significantly different (p < 0.05) from HE/NE group (‡) significantly different (p < 0.05) from HE/HE group (*) Main effect (p < 0.05).

There was a significant interaction between gestational and post-weaning diets for both BK and SNP induced vasorelaxation in the offspring (Figure 1A and B). Specifically, post hoc analysis revealed that the BK and SNP induced relaxation was significantly less in the HE/NE group compared to the NE/NE and the NE/HE groups. The SNP relaxation response for the HE/NE group was also significantly reduced compared to HE/HE, NE/NE, and NE/HE groups.

**Figure 2:**
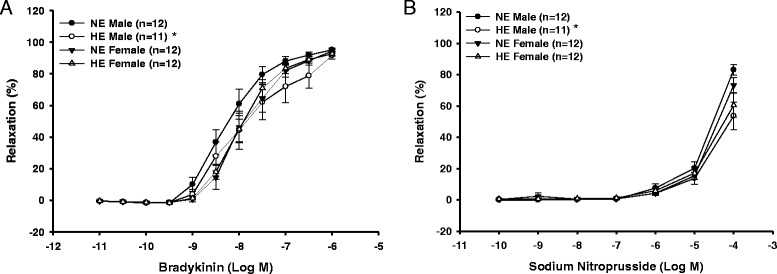
**Endothelium-dependent (A) and endothelium-independent (B) relaxation responses to increasing doses of BK and SNP in the femoral artery of male and female offspring exposed to gestational NE or HE diets, at three months of age, independent of their post-weaning diets.** *Significantly different (p < 0.05) from NE male offspring.

There was also a significant effect of gestational diet on both BK and SNP induced vasorelaxation in the offspring. Specifically, offspring exposed to the HE gestational diet demonstrated significantly less BK and SNP induced vasorelaxation when compared to offspring exposed to the NE gestational diet (Figure 1A and B). These gestational diet induced differences in vasorelaxation responses between HE and NE offspring to BK and SNP were attributed to significant differences in male but not female offspring when examined independently of post-weaning diets (Figure 2A and B). Post-weaning diets had no significant effect on BK or SNP induced vasorelaxation in the femoral arteries of the offspring when examined independently of gestational diets.

## Discussion

The risk of disease development can begin in the intrauterine environment [23]. It is well established that nutrition during the gestational period plays a key role in the genesis of disease in the developing fetus. Specifically, research models of over-nutrition have demonstrated that early dietary insults can alter gene expression and promote atherosclerotic disease development in the developing offspring [24]. Various cell types are known to be involved in the development of atherosclerosis, of which endothelial and vascular smooth muscle cells are the most studied [25]. Poor endothelial cell function is thought to be one of the early markers for the development of atherosclerosis. The results of this investigation in a novel large animal swine model support previous studies reporting that nutrition during the gestational period impacts the vascular function of rodent and non-human primate offspring. In particular, the current findings demonstrate that excess energy intake during pregnancy impairs both endothelial and vascular smooth muscle cell function in the femoral artery of swine offspring. Our results also illustrate that these alterations cannot be impacted by a three-month post-weaning diet intervention.

A small number of studies have investigated how a model of maternal over-nutrition impacts the vascular function of offspring [13-15,17]. Of the few studies that have examined this phenomenon in regards to vascular function, all observed a decrease in endothelium-dependent vasorelaxation in offspring exposed to excess maternal food consumption. The results of the current experiment further support these findings by demonstrating that offspring exposed to a high-energy environment in utero have reduced BK induced endothelium-dependent vasorelaxation. However, this is the first study to demonstrate a sex-specific response with respect to the observed decrease in endothelium-dependent vasorelaxation. Specifically, the male offspring exposed to a gestational HE diet, regardless of their post-weaning diet, had significantly less BK induced endothelium-dependent vasorelaxation when compared to male offspring on the NE prenatal diet (Figure 2A and B). This suggests that the significant differences seen in BK induced vasorelaxation prenatally may be driven by male offspring. Previous studies that examined vascular function have reported responses driven by both male and female offspring [13-15]. In the current investigation, females were not matched for their oestrous cycle, which could have caused these variations [26]. The use of a swine model could be another reason these differences were observed in the current but not previous investigations. All studies to date have investigated maternal nutrition and the vascular outcomes of offspring using a rodent model [13,15,17] except for one utilizing non-human primates [14]. Therefore, the anatomical and physiological differences between these species and swine could influence the observed alterations.

The mechanisms underlying the current findings of reduced BK induced endothelium-dependent vasorelaxation in the offspring of mothers that ingested the HE diet during gestation are currently unclear. One well recognized mechanism for decreased endothelium-dependent vasorelaxation is diminished production of NO by eNOS [27,28]. Of the studies that have observed decreased endothelial function in offspring exposed to a high fat gestational diet, only two have indirectly assessed the NO production through eNOS gene expression [14,17]. Both studies speculated that the decreased function was most likely not attributed to a reduction in NO production since eNOS gene expression was not different between groups [14,17]. However, these findings must be interpreted cautiously since it is well known that differences in gene expression do not always equate to measurable functional outcomes. Differences in NO bioavailability may also contribute to the reported reduction in BK induced vasorelaxation in the offspring of mothers who consumed a HE diet during gestation. It has recently been reported that offspring exposed to a high fat diet during prenatal and/or postnatal life had significantly increased oxygen-derived free radicals [17], which are known to increase the degradation of NO and lead to reduced endothelium-dependent vasorelaxation. It has been suggested that a decreased responsiveness of the offspring’s vascular smooth muscle to NO may play an important role in decreased endothelium-dependent vasorelaxation induced by gestational diet [14]. This is supported by our findings, which demonstrate for the first time that exposure to a HE gestational diet diminishes SNP induced endothelium-independent vasorelaxation in offspring. Lastly, the current findings of reduced BK induced vasorelaxation in the offspring of mothers that consumed a HE diet during gestation may be independent of differences in NO. It is well recognized that endothelial produced prostacyclin (PGI_2_) and endothelial derived hyperpolarizing factor (EDHF) contribute to 75-80% of BK induced vasorelaxation in the in the femoral arteries of swine [21]. Therefore, differences in PGI_2_ and EDHF may contribute to our findings. Future studies will be needed to elucidate the mechanism underlying the current findings.

The current investigation demonstrates that the three-month post-weaning diet does not have an impact on either BK or SNP induced vasorelaxation in offspring. This finding illustrates that the gestational environment is likely a more critical time for the offspring when compared to the early postnatal life. Similar results were observed in a previous investigation examining gestational and suckling diets [15]. It was proposed that gestational exposure to a high fat diet might protect offspring from a high fat dietary insult during suckling [15], which does not hold true during the post-suckling period based on the results from the current experiment. Another study examining the post-weaning diet, however, demonstrated that offspring exposed to a high fat diet prenatally had improved endothelium-dependent vasorelaxation when placed on a control diet post-weaning [14]. However, it is important to note that their offspring were maintained on the post-weaning diet for 3 months longer than those from the current investigation. Therefore, the duration of the post-weaning diet likely contributes significantly to the vascular function outcome. Future studies will be needed to elucidate the critical time periods in which post-weaning diets can influence vascular health.

### Limitations

One of the limitations of the current investigation was our inability to measure gene expression in genes that may influence our functional results. These measurements were attempted for several genes but do technical difficulties with the control genes the results were not reported.

As previously stated, various genes play a role in the NO/GC signaling pathways. Therefore, it is challenging to determine the underlying mechanism for the observed results. Future researchers must thoroughly examine the plethora of genes associated with NO/GC pathway. This may help us better understand the underlying mechanisms behind the reduced endothelium and vascular smooth muscle function. Furthermore the post weaning diet alone did not impact offspring vascular function in the current study. To date, two other investigations have examined the impact of a post-weaning diet on the vascular function of offspring [14,17]. One study found that prenatal and postnatal diets similarly decreased vascular function [17]. Contrary to our findings, the second study demonstrated that a post-weaning diet was able to modify the insults imposed on vascular function by a high-energy gestational diet [14]. The duration of time the animals were maintained on post-weaning diets before vascular analysis was conducted could account for these varied observations. Therefore, the short exposure may not have allowed adequate time for changes to be observed in the offspring’s vascular function in the current investigation.

The lack of vascular dysfunction in offspring that were fed a post-weaning HE diet is inconsistent with studies that have been reported in animals that were fed a high fat or Western style diet [14,15]. We can only speculate that the differences in diet, age, and animal model may contribute to our inability to observe differences in offspring fed a HE versus NE diet. In addition, it is important to note that similarities in weights between NE and HE post weaning fed offspring were similar at 3 months of age and suggest that the offspring on the HE diet naturally reduced their consumption of the HE diet and thus consumed similar calories as the post weaning NE animals. Therefore, similarities in calories consumed may play a role in our inability to detect vascular dysfunction in post weaning HE offspring.

### Clinical implications

Excess dietary intake and lack of physical activity are known to lead to weight gain [3]. However, many women have misconceptions of appropriate weight gain during pregnancy and/or a lack of knowledge [29]. Some women believe that there are no negative implications to weight gain in excess of IOM’s recommendations [29]. Contrary to this perception, the results of the current experiment indicate that gilts consuming the HE diet had greater gestational weight gain when compared to the gilts that consumed an NE diet. Furthermore, vascular function of offspring born to mothers with higher gestational weight gain was diminished when compared to offspring born to mothers with normal gestational weight gain. This illustrates that excess energy intake during pregnancy not only increased maternal weight gain but also had negative implications for the offspring that may lead to increased risk for chronic diseases such as atherosclerosis in later life. Therefore, it is critical for the health of the offspring that physicians educate pregnant women on the importance of weight management during pregnancy.

## Conclusions

The current investigation demonstrates that exposure to a HE diet in utero decreased the BK induced vasorelaxation response in swine offspring. Additionally, the results revealed for the first time that SNP induced vasorelaxation was also diminished in swine offspring that were introduced to the HE diet during gestation. More importantly, our analysis demonstrates that improving the offspring’s post-weaning diet for 3 months could not reverse the damage done during the 4 months of gestation. Therefore, it appears that the vascular function of offspring is impacted to a great extent by the prenatal environment, which may lead to a lifelong disease predisposition that cannot be undone with similar duraton postnatal diet interventions. Consequently, it is critical to inform women about the importance of nutritional intake during gestation and how it directly impacts their offspring vascular function.

## Abbreviations

ANOVA: Analysis of Variance
BK: Bradykinin
EDHF: Endothelial Derived Hyperpolarizing Factor
eNOS: Endothelial Nitric Oxide Synthase
GC: Guanylate Cyclase
HE: High Energy
IOM: Institute of Medicine
NE: Normal Energy
NO: Nitric Oxide
PGF2α: Prostaglandin F2α
SNP: Sodium Nitroprusside
